# Designing a novel and combinatorial multi-antigenic epitope-based vaccine “MarVax” against Marburg virus—a reverse vaccinology and immunoinformatics approach

**DOI:** 10.1186/s43141-023-00575-w

**Published:** 2023-11-28

**Authors:** Bishal Debroy, Sribas Chowdhury, Kuntal Pal

**Affiliations:** 1 Department of Biological Sciences, School of Life Science and Biotechnology, Adamas University, Barasat-Barrackpore Road, Kolkata, West Bengal 700126 India; 2 Department of Biotechnology, School of Life Science and Biotechnology, Adamas University, Barasat-Barrackpore Road, Kolkata, West Bengal 700126 India; 3 Cancer Biology Laboratory, Adamas University, Barasat-Barrackpore Road, Kolkata, West Bengal 700126 India; 4 School of Biosciences and Technology (SBST), Vellore Institute of Technology, Vellore, Tamil Nadu 632014 India

**Keywords:** Marburg virus disease, Immunoinformatics, Epitope, Multi-epitope vaccine, Molecular docking, Molecular dynamics

## Abstract

**Context:**

Marburg virus (MARV) is a member of the Filoviridae family and causes Marburg virus disease (MVD) among humans and primates. With fatality rates going up to 88%, there is currently no commercialized cure or vaccine to combat the infection. The National Institute of Allergy and Infectious Diseases (NIAID) classified MARV as priority pathogen A, which presages the need for a vaccine candidate which can provide stable, long-term adaptive immunity. The surface glycoprotein (GP) and fusion protein (FP) mediate the adherence, fusion, and entry of the virus into the host cell via the TIM-I receptor. Being important antigenic determinants, studies reveal that GP and FP are prone to evolutionary mutations, underscoring the requirement of a vaccine construct capable of eliciting a robust and sustained immune response. In this computational study, a reverse vaccinology approach was employed to design a combinatorial vaccine from conserved and antigenic epitopes of essential viral proteins of MARV, namely GP, VP24, VP30, VP35, and VP40 along with an endogenous protein large polymerase (L).

**Methods:**

Epitopes for T-cell and B-cell were predicted using TepiTool and ElliPro, respectively. The surface-exposed TLRs like TLR2, TLR4, and TLR5 were used to screen high-binding affinity epitopes using the protein-peptide docking platform MdockPeP. The best binding epitopes were selected and assembled with linkers to design a recombinant multi-epitope vaccine construct which was then modeled in Robetta. The in silico biophysical and biochemical analyses of the recombinant vaccine were performed. The docking and MD simulation of the vaccine using WebGro and CABS-Flex against TLRs support the stable binding of vaccine candidates. A virtual immune simulation to check the immediate and long-term immunogenicity was carried out using the C-ImmSim server.

**Results:**

The biochemical characteristics and docking studies with MD simulation establish the recombinant protein vaccine construct MarVax as a stable, antigenic, and potent vaccine molecule. Immune simulation studies reveal 1-year passive immunity which needs to be validated by in vivo studies.

**Supplementary Information:**

The online version contains supplementary material available at 10.1186/s43141-023-00575-w.

## Background

The severe, acute, and recurring Marburg virus disease (MVD) is caused by the Marburg virus (MARV) and has been linked to many devastating outbreaks with fatality rates going as high as 83–88%. Reports of the first known outbreak were described in 1967 in the cities of Marburg in Germany and Belgrade in Serbia, leading to the detection of the disease. This was followed by several outbreaks of the fatal disease that recurred over subsequent periods with the most recent outbreak reported in Ghana (2022), Tanzania, and Equatorial Guinea (2023). The most fatal endemic was the MVD outbreak in Angola in 2004–2005 which had 252 cases reported and 227 confirmed deaths inferring a 90% fatality rate [1]. MARV is related to the hemorrhagic fever-causing Ebola virus (EBOV). Due to its non-segmented, negative-strand RNA genome serving as a genetic template for reproduction, the virus is categorized in the order of mononegavirales [2, 3]. The Egyptian fruit bat, *Rousettus aegyptiacus*, is the reservoir species for MARV, and its rare outbreak is mostly related to the geographical range of these bats. Prior research revealed that the human population is in significant danger from the MARV infection of the *Rousettus* bats, which may climb up to 10% in young bats during seasonal surges [4]. According to some reports, the virus may also exist in pigs, African green monkeys, and other susceptible reservoirs [1, 5, 6].

**Fig. 1:**
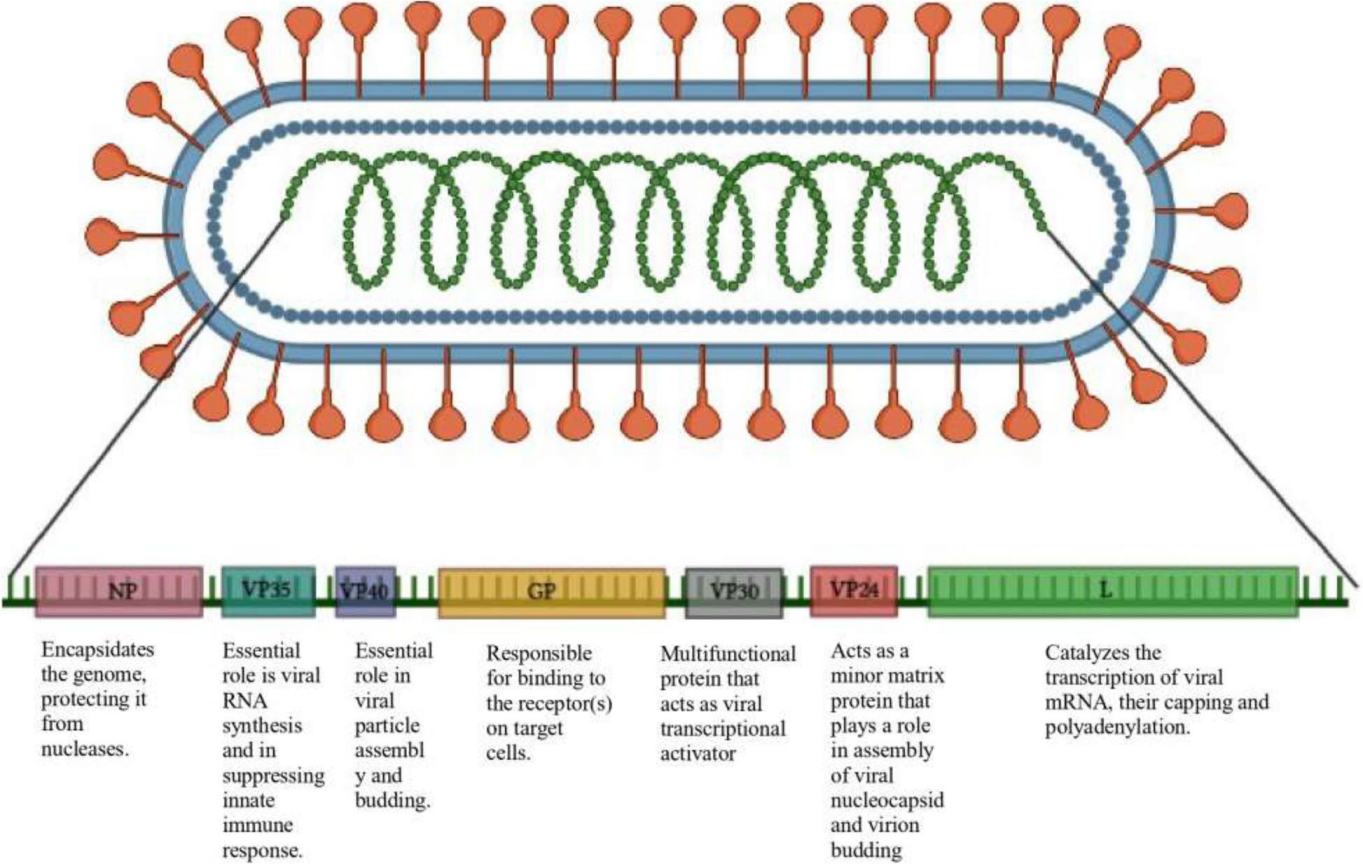
Genomic organization of Marburg virus representing the function of structural and non-structural proteins, created with BioRender (https://www.biorender.com/)

The most virulent version of this pleomorphic virus measures 80 nm in diameter and 790 nm in length [7–9]. As shown in Fig. 1, this mononegavirales has a 19.1 Kb length RNA that codes for seven structural proteins, including nucleoprotein (NP), glycoprotein (GP), viral protein 24 (VP24), viral protein 30 (VP30), viral protein 35 (VP35), viral protein (VP40), and large protein (L) [9–11]. The nucleocapsid complex is made up of the proteins NP, VP30, VP35, and L, where L functions as RNA-dependent RNA polymerase (RdRp) and VP35 is a co-factor of polymerase as well as an IFN antagonist [9, 12]. GP is required for the virus to adhere to its host cell [13]. VP40 interferes with the JAK-STAT pathway and is linked to the virus’s budding, whereas VP24 is responsible for the progeny virion’s release from the host cell [9, 14, 15]. Despite some findings showing the presence of viral particles in the rectal, mouth, and urine samples of the MARV-infected bats, transmission of the virus from the reservoir to humans is yet to be known [9, 16]. Further research revealed that the virus is present in an infected bat’s intestine, lung, kidney, salivary gland, and reproductive systems, which raises the possibility of both vertical and horizontal transmission of the virus [17]. The blood, body fluids like saliva and breast milk, and sexual contact are all possible routes for human-to-human transmission [9].

The virus can enter the host in several different ways, and after cellular attachment to the TIM-I receptor, endocytosis, and fusion, it releases its viral RNA into the host cell [2, 9]. The MARV VP40 interacts with the viral nucleocapsid complex and serves as an interface for both filopodia and sub-viral MARV particles. Filopodia are in intimate contact with the adjacent cells, which promotes the spreading of MARV and raises the viral titer in the blood of infected humans [2, 18]. The liver assumes a pivotal role in MARV replication, leading to hepatocyte degradation, reticuloendothelial system impairment, and hepatocyte injury via a measured inflammatory cascade, resulting in edema and significant damage to the infected host’s system. Primary infection-mediated immunomodulation decreases the proliferation of immune cells that cause secondary infections [2, 9]. The liver damage in MARV-infected patients is more severe than in the case of EBOV infection, with the lymph nodes, spleen, testes, ovaries, gastrointestinal system, and endocardium suffering from severe necrotic lesions [2].

The virus has three stages of infection and an incubation period of 2 to 21 days [1, 9]. Phase 1, also known as the generalized phase, lasts for 5 days and is characterized by a high fever (39 to 40 °C) and influenza-like illness. Fatigue, dysphagia, pharyngitis, leukopenia, and thrombocytopenia are other manifestations [6, 9, 19]. Phase 2 continues with a high fever and then progresses to liver, pancreatic, and renal dysfunction. About 75% of the patients experience hemorrhagic signs, along with neurological symptoms, dyspnea, and abnormal vascular permeability [9, 20]. Phase 3, the final stage, might result in either of two outcomes. The patient may enter the phase of recovery or the infection could become lethal. Fatality is characterized by shock and multi-organ failure which is the primary cause of death [21]. A severe metabolic imbalance takes place, leaving a negative impact on a patient’s health. During this stage, it is common to experience exhaustion, partial amnesia, sweating, peeling skin in rash-affected areas, and secondary infections [9].

Classification of MARV as priority pathogen A by The National Institute of Allergy and Infectious Diseases (NIAID) and its categorization as category A bio-terrorism agent by the Centers for Disease Control and Prevention (CDC) necessitates the need of a vaccine candidate with long-term adaptive immunity [22]. There is currently no commercialized vaccine or known cure targeted towards combating MVD. The previously reported vaccines against MARV are mostly based on certain exogenous proteins, which were found to be susceptible to significant rates of mutation [2, 23]. In light of the COVID-19 pandemic, it is clear that a high number of hypervariable regions in surface-exposed viral proteins is related to increased viral pathogenicity and decreased efficiency of vaccines in conferring long-term immunity against viral infection [24]. Hence, developing a vaccine which is not only antigenic and inclusive of epitopes of all antigenic viral proteins but also addresses the long-term stability of the vaccine construct is the need of the hour.

In this study, we have used computational biology and immunoinformatics to design a multi-epitope vaccine that is stable and non-allergic to humans while yet having the capacity to trigger the required immune response against the Marburg virus. We have used B-cell and T-cell epitopes of GP, VP24, VP35, VP40, and RdRp to design a combinatorial vaccine with the ability to generate a strong immune response while also conferring stable, long-term immunity. The viral protein VP24 is in charge of the release of new viral offspring, and the surface glycoprotein, or GP, facilitates the viral entry in the host cell. By altering the JAK-STAT pathway, immune gene suppression, and IFN-driven cascade, VP35 and VP40 play a part in the host system’s immune evasion. RdRp, out of all of them, is crucial for viral replication since it replicates the viral genome. All these factors establish the epitopes of these proteins as suitable antigenic targets for the development of an efficient vaccine construct.

Based on our analysis of the immunogenicity and conserved regions of epitopes of the five crucial viral proteins, we designed a combinatorial multi-epitope protein as a potential vaccine construct. The binding stability and molecular interactions were checked by docking with Toll-like receptors TLR2, TLR4, and TLR5 and molecular dynamic studies further confirmed the stability of vaccine construct-receptor complex. Finally, immune simulation results demonstrated a human immune response to the vaccine construct, which was found to confer reliable short-term and long-term immunity. As the outcome of this study, we hereby predict MarVax as a highly antigenic and stable multi-epitope vaccine against the Marburg Virus and can be further studied and validated using in vitro and in vivo models.

## Methods

### Retrieval of the primary data

The primary information about the five essential proteins encoded by the MARV genome, including the glycoprotein (GP) (UniProtKB ID: P35253), RNA-dependent RNA polymerase (RdRp) (UniProtKB ID: P31352), viral proteins VP24 (UniProtKB ID: P35256), VP35 (UniProtKB ID: P35259), and VP40 (UniProtKB ID: P35260), were retrieved from UniProtKB (https://www.uniprot.org/). The Protein Data Bank from the RCSB server (https://www.rcsb.org/) was used to retrieve the protein structures for GP (PDB ID: 5UQY), VP25 (PDB ID: 4OR8), and VP40 (PDB ID: 5B0V). Robetta (https://robetta.bakerlab.org/) [25–33] and Swiss Model (https://swissmodel.expasy.org/) [34] were used respectively, to model the protein structures of RdRp and VP35. Both the servers perform homology modeling of query protein using a suitable protein as a template which is having high sequence homology, and structure is available in RCSB-PDB. Lastly, the best-modeled structure of the RdRp and VP35 generated by the servers was subjected for structural validation by utilizing the web server called PROCHECK (https://saves.mbi.ucla.edu/) [35, 36]. The conformational stability of modeled structures was analyzed by evaluating the Ramachandran Plot for RdRp and VP35. Finally, each protein model was edited in PyMOL ver2.4 and prepared for further studies.

### T-cell and B-cell epitope prediction, screening, and sorting

All the five proteins namely GP, RdRp, VP24, VP35, and VP40 were predicted for the presence of T-cell epitopes. The TepiTool server (http://tools.iedb.org/tepitool/) [37–42] of the IEDB analytical resource tool was used to predict MHC class I restricted epitopes for GP, RdRp, VP24, VP35, and VP40. The list of representative alleles from various HLA super-types was selected, and the percentile rank was set to the value of one for the prediction of MHC class I epitopes. The same server was also used for the prediction of MHC class II-restricted epitopes for all of these five proteins. For this prediction, a pre-selected panel of alleles covering the three human MHC class II isotypes which are HLA-DR, HLA-DQ, and HLA-DP was used, and percentile rank 10 was chosen. After screening the predicted epitopes based on percentile rank, peptide length, and conservancy levels, the top five epitopes for both MHC class I and class II were chosen for docking.

Similarly, the five MARV proteins were predicted for the presence of B-cell epitopes using the IEDB server ElliPro (http://tools.iedb.org/ellipro/) [43]. This program predicted both linear and discontinuous epitopes. For the development of a multi-epitope vaccine, a linear epitope is the best choice. Thus, the 3D structure of the protein was uploaded to this web server in PDB format. By averaging the protrusion index (PI) values over the residues of the anticipated epitopes, ElliPro assigns a score to each predicted epitope. The parameter was fixed by limiting the maximum amino acid length to ten residues and the minimum score of 0.5. The top five predicted linear epitopes were chosen for docking after being filtered based on PI score, peptide length, and conservancy levels.

### BLAST and multiple sequence alignment

Based on the conservation level of each protein sequence as assessed by BLAST in NCBI, five MARV strains were chosen. The five strains were the Musoke strain (1980) and Ravn strain (1987) from Kenya, Ozolin strain (1975) from South Africa, Popp strain (1967) from West Germany, and Angola Strain (2005). A multiple-sequence alignment tool ClustalOmega was used to align the five protein sequences of the chosen strains. The aligned sequences were rendered using ESPript3.0 (https://espript.ibcp.fr/ESPript/ESPript/), which highlighted the conserved regions of the protein.

### Sorting of epitope and designing of the protein vaccine

The first step in designing a protein vaccine was to screen the predicted T-cell epitopes and B-cell epitopes present in the five MARV proteins. The T-cell epitopes predicted using the TepiTool web server were filtered based on the percentile rank and peptide length of the epitopes. In the same manner, the linear B-cell epitopes predicted using the Ellipro web server were filtered on the basis of the PI score and peptide length of the epitopes. Further, the filtered epitopes were sorted based on the alignment which was performed using ESPript3.0 to identify the conserved regions in the amino acid sequence of the proteins.

Based on the screened T-cell and B-cell epitopes, and the most conserved sequence found from the alignment, the best five T-cell and B-cell epitopes for all the five MARV proteins were sorted and a docked with TLRs to analyze the interacting property of these epitopes.

### Docking analysis with Toll-like receptors (TLRs)

Toll-like receptors or TLRs are a family of proteins found on the surface of many cells, including the immune cells such as the macrophage and dendritic cells and they recognize antigens associated with the pathogen. In general, TLR1, TLR2, TLR4, TLR5, and TLR6 are the most commonly expressed TLRs in human cells. Among this cohort, TLR2, TLR4, and TLR5 are particularly significant.

TLR1 and TLR6 form heterodimers with TLR2 which is expressed on the surface of many cells, including monocytes, macrophages, dendritic cells, and endothelial cells. Similarly, TLR4 is also expressed on the surface of macrophages, dendritic cells, and epithelial cells. Both of them can recognize bacterial lipopolysaccharide (LPS). On the other hand, TLR5 is expressed on the surface of cells such as intestinal epithelial cells, and it can recognize flagellin, a component of bacterial flagella.

Based on the above facts, three Toll-like receptors (TLRs), TLR2 (PDB ID: 3A79), TLR4 (PDB ID: 3FXI), and TLR5 (PDB ID: 3J0A) were obtained from PDB. Using the server MDockPeP (https://zougrouptoolkit.missouri.edu/mdockpep/), all of the chosen T-cell and B-cell epitopes of the five MARV proteins were initially docked with these three TLRs. This web server, for protein-peptide docking, models the peptide first, then globally and flexibly examines protein-peptide binding patterns, and lastly rates and scores the observed binding modes [44, 45].

### Secondary structure prediction and molecular docking

Five of the best epitopes from both T-cells and B-cells were chosen based on the initial docking of all the selected peptides from the five proteins with the three TLRs. A GSAGSAGSA linker was then used to link these selected peptide sequences, and the synthetic protein was modeled in the Robetta server. The PROCHECK (https://saves.mbi.ucla.edu/results?job=1247870&p=procheck) and PSIPRED (http://bioinf.cs.ucl.ac.uk/psipred/) [46, 47] web servers were used to validate the predicted structure. Using the internet server Galaxy Refine (https://galaxy.seoklab.org/cgi-bin/submit.cgi?type=REFINE) [48, 49], the protein was optimized. Only one of the five models created by Galaxy Refine was chosen based on the structural validation by PROCHECK. After that, the chosen protein model with the best score was docked again with the three TLRs (TLR2, TLR4, and TLR5) using the internet server ClusPro2.0 (https://cluspro.bu.edu/login.php) [50, 51].

### Physiological property prediction of the multi-epitope protein

Alongside protein-TLR docking, the physiological properties of the modeled protein were evaluated. Using ProtParam, Scratch Protein Predictor, and NetChoP, the various vaccine parameters were assessed, including stability, molecular weight, isoelectric point (pI), antigenicity, solubility, and solvent accessibility. The ProtParam tool (https://web.expasy.org/protparam/) [52] provides theoretical information regarding the physiochemical properties of the protein, including stability, molecular weight, pI, Grand Average of Hydrophobicity (GRAVY) index, and others. The Scratch Protein Predictor site (http://scratch.proteomics.ics.uci.edu/) [53] contains a subset of tools, including ACCpro and ACCpro2.0 for solvent accessibility, SOLpro for solubility, and the ANTIGENpro for antigenicity evaluation. The Proteasomal Cleavage Prediction tool of IEDB analytical resources (http://tools.iedb.org/netchop/) [54, 55] provides information about the proteasomal decay of the protein in graphical format. The data was cross-validated using DTU Healthtech’s NetChop (https://services.healthtech.dtu.dk/service.php?NetChop-3.1), which generates neural network predictions for the cleavage sites of the human proteasome. To estimate the characteristics of this protein, we used the default parameters of these websites.

### Molecular dynamics simulation and immune simulation

Protein in water molecular dynamics (MD) simulation of the protein-TLR5 complex was carried out using the internet server WebGro (https://simlab.uams.edu/) [56, 57] for the evaluation of binding stability, conformation changes, and interaction modes. For the simulation, the PDB file of the protein-TLR5 complex was uploaded and the simulation was performed by applying GROMOS96 43a1 force field. The complex structure was solvated in a triclinic box with a simple point charge (SPC) water model. Then, 0.15M NaCl was added as a neutralizer in the simulation system. The simulation was allowed to run for 50 ns in the NPT equilibrium type with a constant pressure (1.0 Bar) and temperature (300 k). One thousand frames per simulation was generated to determine the root mean square deviation (RMSD), root mean square fluctuation (RMSF), radius of gyration (Rg), solvent-accessible surface area (SASA), and average number of H-bonds in each frame.

Molecular dynamics (MD) simulation to determine the root mean square fluctuation (RMSF) of the vaccine construct-TLR5 complex was carried out using the internet server CABSflex2.0 (http://biocomp.chem.uw.edu.pl/CABSflex2). The cycle count was set to 50, whereas all other parameters were kept the same.

At last, the designed vaccine was also run through with an immune simulation using the C-ImmSim online web server (https://kraken.iac.rm.cnr.it/C-IMMSIM/) [58, 59] to evaluate how well this foreign antigenic protein would influence the immune response of the human host. This simulation was done targeting the A0101 and B0702 HLA alleles of MHC class I and the DRB1_0101 and DRB1_0701 HLA alleles of MHC class II. Ten microliters of simulation volume containing 1000 antigenic particles without lipopolysaccharide (LPS) was injected for analyzing the host’s immune response for 350 days by setting simulation steps to 1050 (1 step = 8 h in real life).

## Results

### Conservation check and epitope selection

The highly conserved portions of a protein become a suitable target for the vaccine designed for a virus that is evolving. The conserved regions of the essential viral proteins were identified using sequence alignment in ESPript3. The most conserved proteins were found to be VP35 and VP40, followed by VP24, RDRP, and GP which exhibited the least level of conservation.

Using the TepiTool web server, T-cell specific epitopes were predicted which gave the list of T-cell epitopes for each of the five essential MARV proteins with percentile ranks ranging from 1 to 0.01. Similarly, B-cell-specific epitopes predicted using the ElliPro web server generated a list of epitopes with scores based on the protrusion index (PI) value and located linear B-cell epitopes with PI values ranging from 1.0 to 0.5.

Five epitopes for each T-cell MHC class I and class II and B-cell were selected after the predicted epitopes were screened based on peptide length, sequence conservation, and prediction scores. The selected epitopes are listed in the Supplementary tables (1), (2), and (3). All the selected epitopes were then employed for docking analysis with three TLRs.

### Molecular docking analysis

The three TLRs were used for initial docking with the chosen antigenic peptides, which are listed in Supplementary tables (1), (2), and (3). For each submission, the docking process generates ten docking models, with the best model selected based on the binding energy. A total of 2250 docked models were projected, of which the binding energies of the top 225 docking models for T-cell and B-cell combined, ranging from − 12.1 to − 6.2 kcal/mol and from − 13.5 to − 6.0 kcal/mol, respectively, were recorded. Based on the lowest binding energy, five docked models both from T-cell and B-cell including all of the MARV proteins and three TLRs were selected from a total of 225 peptide-TLR complexes. Table 1 lists the selected dock complexes alongside their peptide sequences and binding energies.

**Table 1:** Binding energy of the selected T-cell and B-cell epitopes

	Peptide-TLR complex	Peptide sequence	Binding energy (kcal/mol)	HLA allele
T-cell	VP24-TLR2	^118^IVHMLSEWLLLEVTS^132^	− 9.6	HLA-DRB1*01:01
	GP-TLR5	^579^EERTFSLINRHAIDF^593^	− 9.1	HLA-DRB1*07:01
	RDRP-TLR4	^366^QQYCELFSLQKHWGH^380^	− 11.5	HLA-DRB1*11:01
	VP35-TLR5	^1^MWDSSYMQQVSEGLM^15^	− 12.1	HLA-DQA1*05:01 HLA-DQB1*02:01
	VP40-TLR5	^112^QFTHNGQKFVRVNRL^126^	− 9.3	HLA-DRB3*02:02
B-cell	GP-TLR5	^548^LIKNQNNLVCRLRRLANQ^565^	− 10.9	---
	RDRP-TLR4	^1115^FLRAYSWSDVLKGKRL^1130^	− 13.5	---
	RDRP-TLR5	^1115^FLRAYSWSDVLKGKRL^1130^	− 9.8	---
	VP35-TLR2	^208^LSAKDLALLLFTHLPGNNT^226^	− 10.9	---
	VP24-TLR2	^104^DQELQQSLIPGFRSIVHM^121^	− 11.6	---

### Multi-epitope vaccine construction and structure modeling

A multi-epitope vaccine MarVax was created by assembling the selected epitopes listed in Table 1 and additional regions on both N- and C-termini to maintain the local fold of the region. The GSAGSAGSA linker has been used to link the epitopes one after the other. The glycine-serine-alanine (GSA) is a preferred amino acid sequence to utilize as a linker to connect two peptides. In general, polar uncharged or charged residues are the preferred amino acids [60]. They should also be flexible to allow the adjoining protein domains to fold and move properly with respect to one another. According to studies by Agros [61] and George and Heringa [62], glycine, serine, and alanine had propensities of 1.25, 1.46, 1.05, and 0.835, 0.947 and 0.964, respectively. The result greater than 1 (> 1) denotes that these amino acids are found in high numbers in the linker. Three repeats of GSA serve to prevent steric hindrance between the peptides and also give flexibility together with glycine and serine. The generated protein sequence is:

MGSAGSAGSAGSALRIWSVQEDDLAAGLSWIPFFGPGIEGLYTAVLIKNQNNLVCRLRRLANQTAKSLELLLRVTTEERTFSLINRHAIDFLLTRWGSAGSAGSATCTVDVANFLRAYSWSDVLKGKRLIGAGSAGSAGSANLSAKDLALLLFTHLPGNNTPFHILAQVLSKIAYKSGKSGGSAGSAGSAEPFLALRILLGVALKDQELQQSLIPGFRSIVHMLSEWLLLEVTSAIGSAGSAGSAKLTPQQYCELFSLQKHWGHPVLYGSAGSAGSAMWDSSYMQQVSEGLMTGKGSAGSAGSSYTITQFTHNGQKFVRVNRLG

**Fig. 2:**
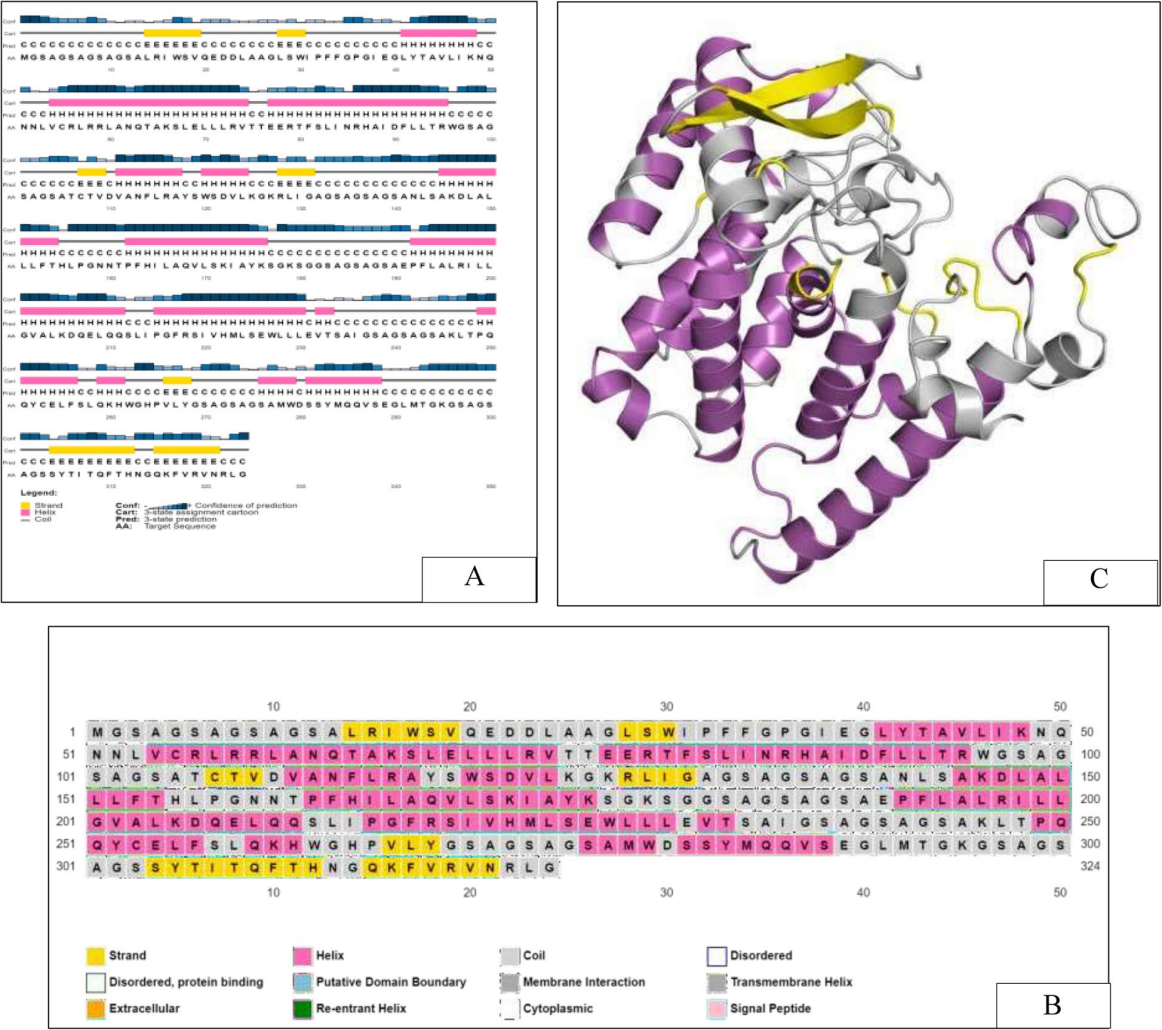
Structural organization of multi-epitope vaccine molecule “MarVax.” (**A**) PSIPRED predicted secondary structure organization with confidence prediction. (**B**) Distribution of the secondary structure within the vaccine molecule. (**C**) Three-dimensional conformation of recombinant vaccine “MarVax”, modeled using Robetta web server

Robetta was used for *ab-initio* modeling of the multi-epitope vaccine construct MarVax, and GalaxyRefine was used to refine the modeled vaccine construct. Additionally, the modeled structure was validated using two servers namely PROCHECK and PSIPRED. Figure 2 shows the secondary structure organization, as well as the 3D conformation of the designed protein vaccine, complementing the PSIPRED predicted data.

The Ramachandran plot of the modeled protein vaccine was generated using the PROCHECK web server and showed 97.8% residues residing in allowed regions, while 2.2% residues are in disallowed regions. The Ramachandran plot further exhibited 248 residues in the most favored region and six residues in disallowed regions, thereby supporting the correctness of the molecular conformation. The Z-score of the constructed multi-epitope protein was calculated using the PROSA server (https://prosa.services.came.sbg.ac.at/prosa.php) which came out to be 7.98, indicating that the vaccine construct is structurally stable. The Ramachandran plot and Z-score plot of the modeled vaccine construct are given in Supplementary Figure 3A and 3B, respectively. The distribution of the B-cell and T-cell epitopes in the protein is illustrated in Supplementary Figure 1. The T-cell epitopes are colored blue, whereas the B-cell epitopes are colored red. The modeled protein has all of its epitopes on the surface, which makes it a suitable antigenic protein that immune cells can detect. The three TLRs were once more docked with this modeled protein vaccine to examine its binding properties.

### Efficacy analysis of recombinant vaccine MarVax by docking with TLRs

To evaluate the immunogenic ability of the vaccine molecule, the modeled multi-epitope protein was docked with the three TLRs. The web server ClusPro2.0 was used to conduct this protein-protein docking. The docking of this multi-epitope protein with the three TLRs produced a total of 30 models as each submission yielded 10 models. The top three models, one from each TLR, were chosen after these models were screened based on their lowest weighted score. As shown in Table 2, this provided us with information indicating that the MarVax-TLR5 complex has the least negative binding score, followed by the MarVax-TLR4 complex and the MarVax-TLR2 complex.

**Table 2:** Binding energy scores of protein-TLR complex

Complex	Binding energy (kcal/mol)
Protein-TLR2	− 18.2
Protein-TLR4	− 19.8
Protein-TLR5	− 20.8

**Fig. 3:**
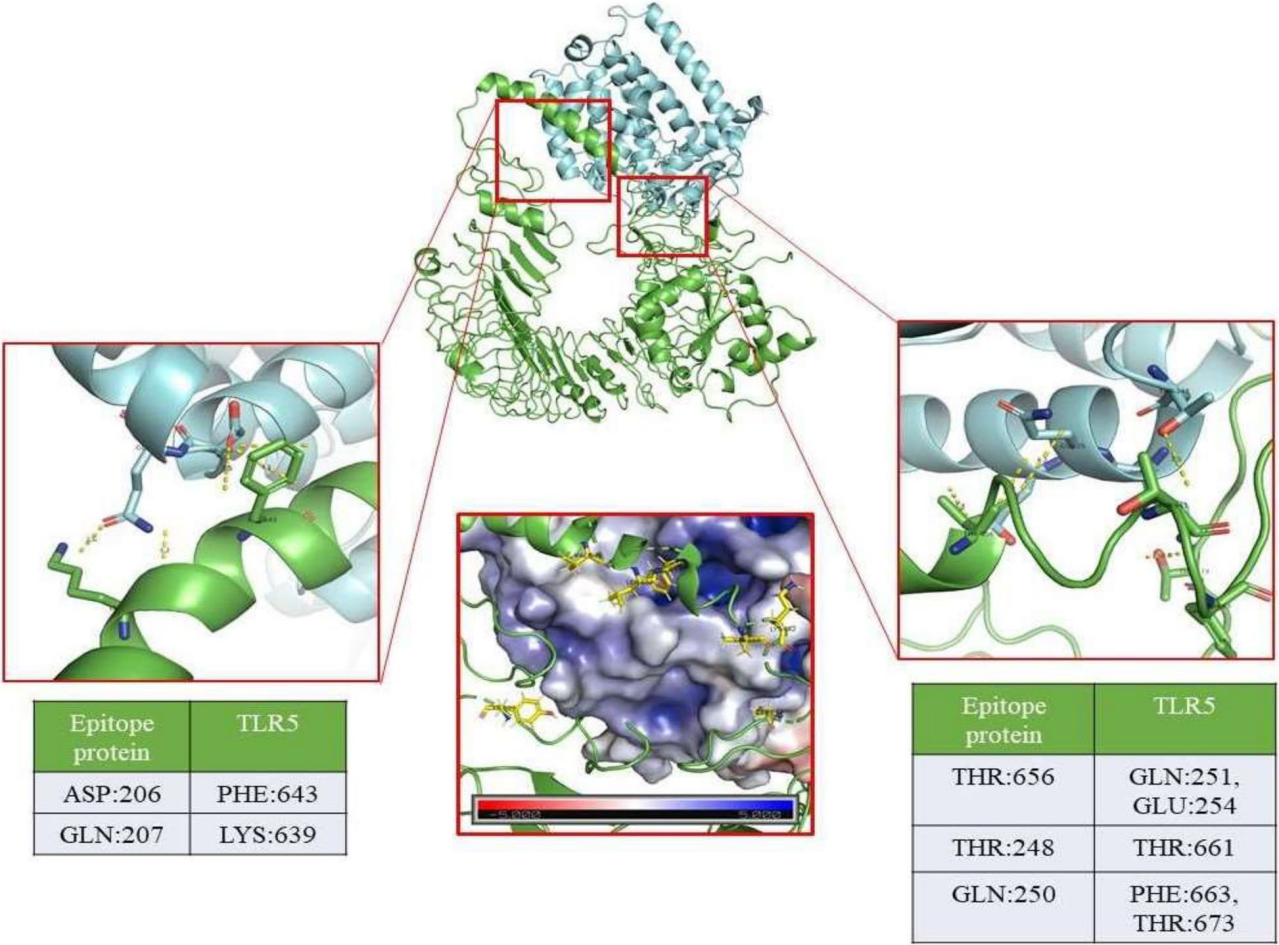
Molecular interaction of recombinant vaccine MarVax-TLR5. TLR5 is highlighted in green color; the MarVax in cyan color. The electrostatic interaction between the vaccine construct and TLR5 was developed by the APBS wizard. Image generated using the PyMOL Molecular Graphics System, Version 2.4, Schrödinger, LLC

MarVax-TLR5 complex is stabilized by eight potential hydrogen bonds between Asp:147 and Val:648, Asn:142 and Leu:652, Ala:141 and Phe:653, Ala:138 and Val:660, Glu:39 and Lys:662, Ile:38 and Lys:662, Ser:137 and Cys:670, and Tyr:175 and Tyr:609 as shown in Fig. 3. The complex’s electrostatic analysis revealed the presence of hydrophobic and charged residues close to the interacting region. This suggests the possibility of potential hydrophobic interactions, weak Van der Waals interactions, and Pi-interactions. Supplementary Figures 2A and 2B further show the interaction of the vaccine complex with TLR 2 and TLR 4.

### Vaccine property analysis

The modeled recombinant vaccine consists of 324 amino acids with 34 kDa molecular weight. The Global distance test-high accuracy (GDT-HA) scores, which range from 0 to 1, are the overall indicators of how well a predicted model matches the experimental structure (with one corresponding to a maximum accuracy) [63, 64]. The GDT-HA value of this protein is 0.9877, which indicates the correctness of the model. The isoelectric point of the recombinant vaccine is 9.49, and its predicted antigenicity is 0.520688. The hydrophobicity of a protein is denoted by the grand average of hydropathicity index (GRAVY), which calculates the sum of the hydropathy values of all the amino acids divided by the length of the sequence. The greater protein solubility is associated with a lower GRAVY index. GRAVY index of MarVax was estimated to be 0.09. Proteins with a hydrophobicity score (arbitrary unit) less than 0 are more likely to be globular (hydrophilic), while proteins with a score greater than 0 are more likely to be membranous (hydrophobic) [65]. DeepTMHMM server (https://dtu.biolib.com/DeepTMHMM) was used to predict the transmembrane helices of the protein which showed that the protein had no transmembrane helices. Both these GRAVY index values and trans-membrane predictions validate the globular three-dimensional model of the vaccine. To check the nobility of the vaccine, its sequence was analyzed for structural homology with humans using pBLAST which showed 0% similarity with any human protein, thereby ruling out any possibility of autoimmune stimulation. Half-life of the vaccine was estimated to be 30 h with about 110 Proteasomal cleavage sites. Table 3 lists the physicochemical characteristics of the designed protein vaccine.

**Table 3:** Physicochemical properties of the multi-epitope protein

Property	Value
Total amino acids	324
Molecular weight	34 kDa
GDT-HA	0.9877
Rama favored	94.1%
Clash score (C-score)	14.0
pI	9.49
Antigenicity	0.520688
GRAVY Z-score	0.09 7.98
Proteasomal cleavage site	110
Estimated half-life (human reticulocytes)	30 h

### Molecular dynamics simulation and immune simulation study

**Fig. 4:**
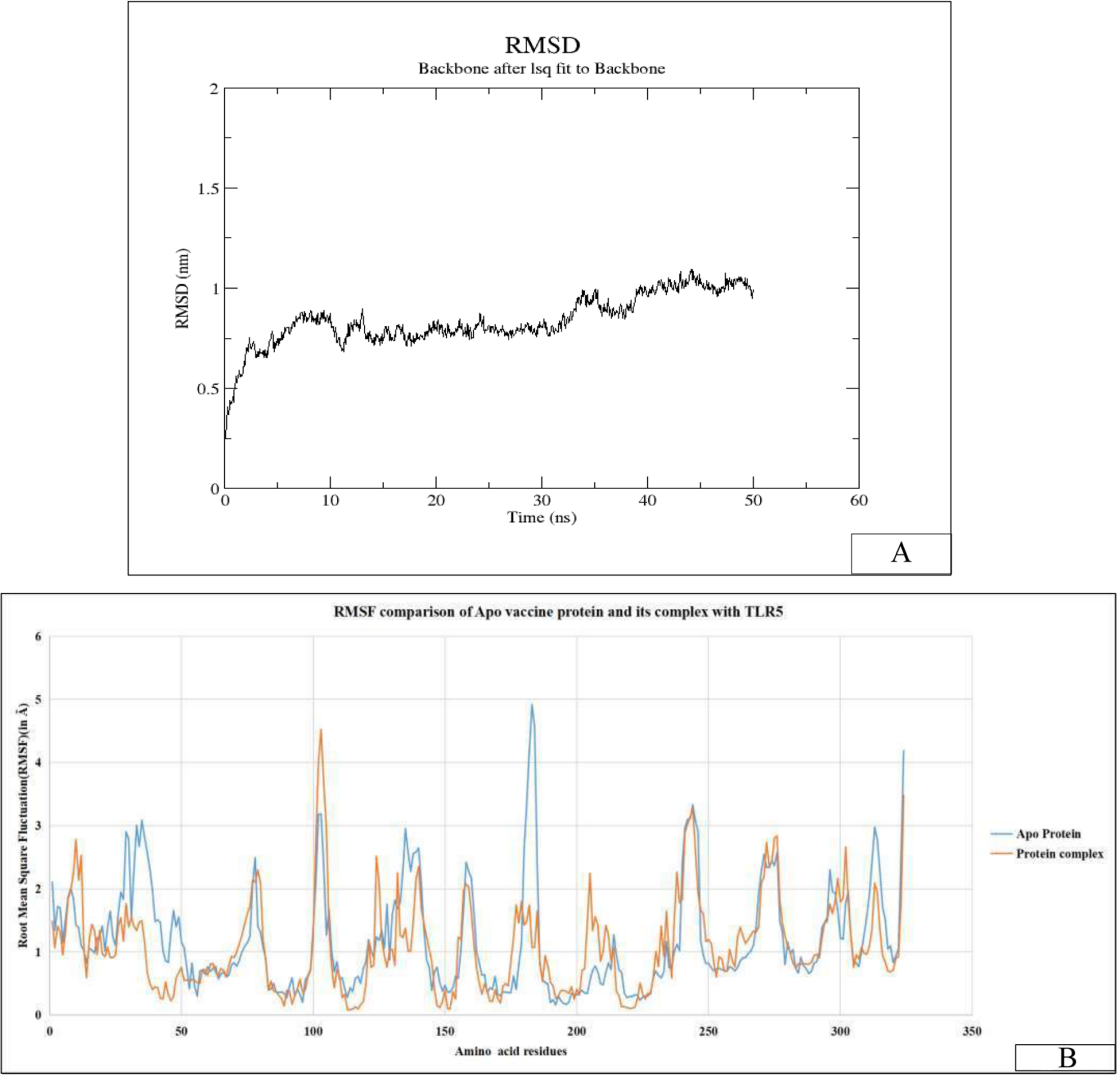
Molecular dynamics analysis of vaccine molecule MarVax-TLR5 complex. (**A)** RMSD analysis of the MarVax-TLR5 complex for 50 ns (**B**) RMSF analysis of Apo vaccine (blue) and MarVax-TLR5 complex states (orange) showed significant stabilization in many regions

The molecular dynamics simulation was performed for 50 ns to understand the dynamics stability associated with the complex formation of the vaccine molecule with TLR5. The simulation gave the RMSD of the complex, which when analyzed; it was found that the bound complex is stable with a very small deviation. The interactions were found to be stable between the vaccine and the TLR5 within this time frame. The RMSD graph of this molecular dynamics is illustrated in Fig. 4A. The average Radius of Gyration (R_g_) value of the MarVax-TLR5 complex was 3.75 nm during this 50 ns simulation. The solvent-accessible surface area (SASA) of the protein-protein complex was estimated to be 525 nm^2^.

To understand the stabilization of vaccine molecule upon complex formation in terms of RMSF, a MD simulation of the protein complex was performed. The comparable RMSF results were obtained by running the simulation of the apo-protein and the complex protein independently. The fluctuations in the amino acid residues are represented in the form of the graph shown in Fig. 4B. The vaccine molecule in the protein-TLR5 complex is found to be stable and exhibits little atomic fluctuation. Additionally, TLR5 is also structurally stable following its interaction with the multiple-epitope protein, validating the structural integrity of the MarVax-TLR5 complex.

**Fig. 5:**
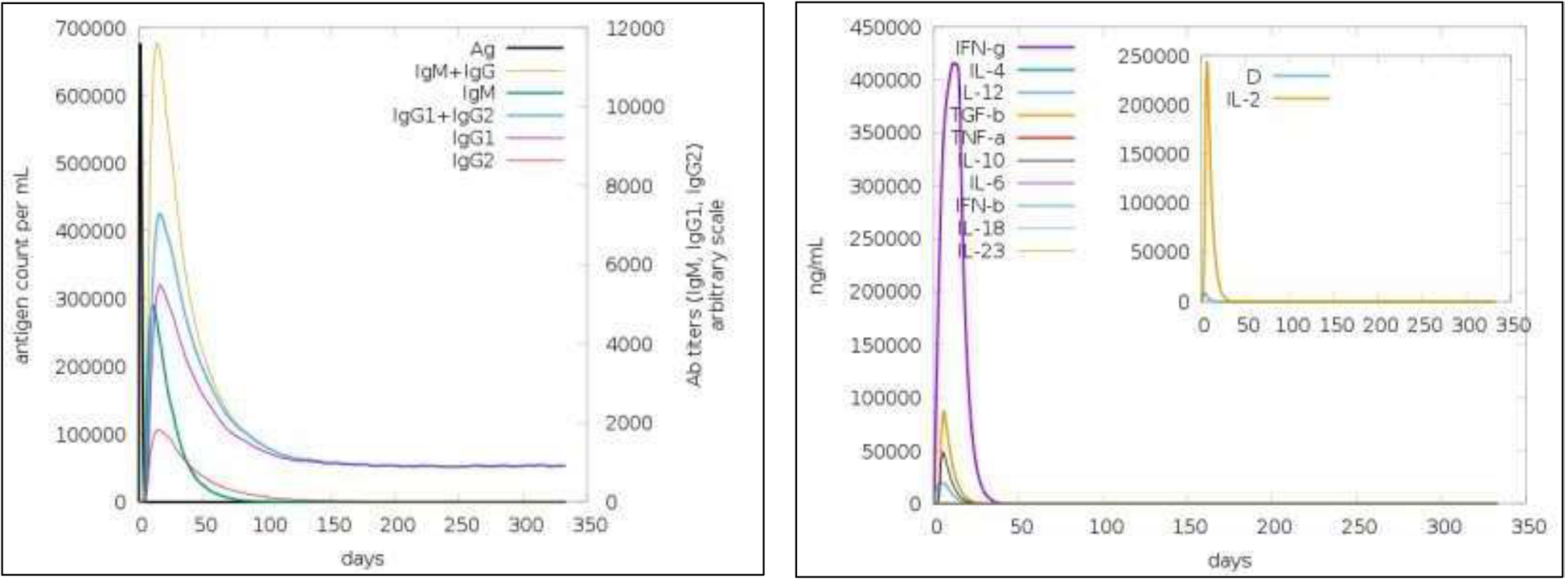
Production of immunoglobulin (Ig) and cytokines against the immunogenic vaccine molecule MarVax

The immune simulation of the recombinant vaccine molecule was performed, which demonstrated a high IgG, IgM, IFN-γ, and IL-2 production upon vaccination, as shown in Fig. 5. Prominently, IgG and IgM were present in blood serum with a peak in the first 30 days and then gradually decreased. IFN-γ remained at a high peak for 30 days, followed by a progressive decrease. This denotes that the vaccine molecule confers an active immunity lasting for almost 2 months. Following vaccination, there was a rise in the population of both cytotoxic T-cells and natural killer cells. Furthermore, B-cell, helper T-cell, cytotoxic T-cell, and natural killer (NK) cell production increased.

**Fig. 6:**
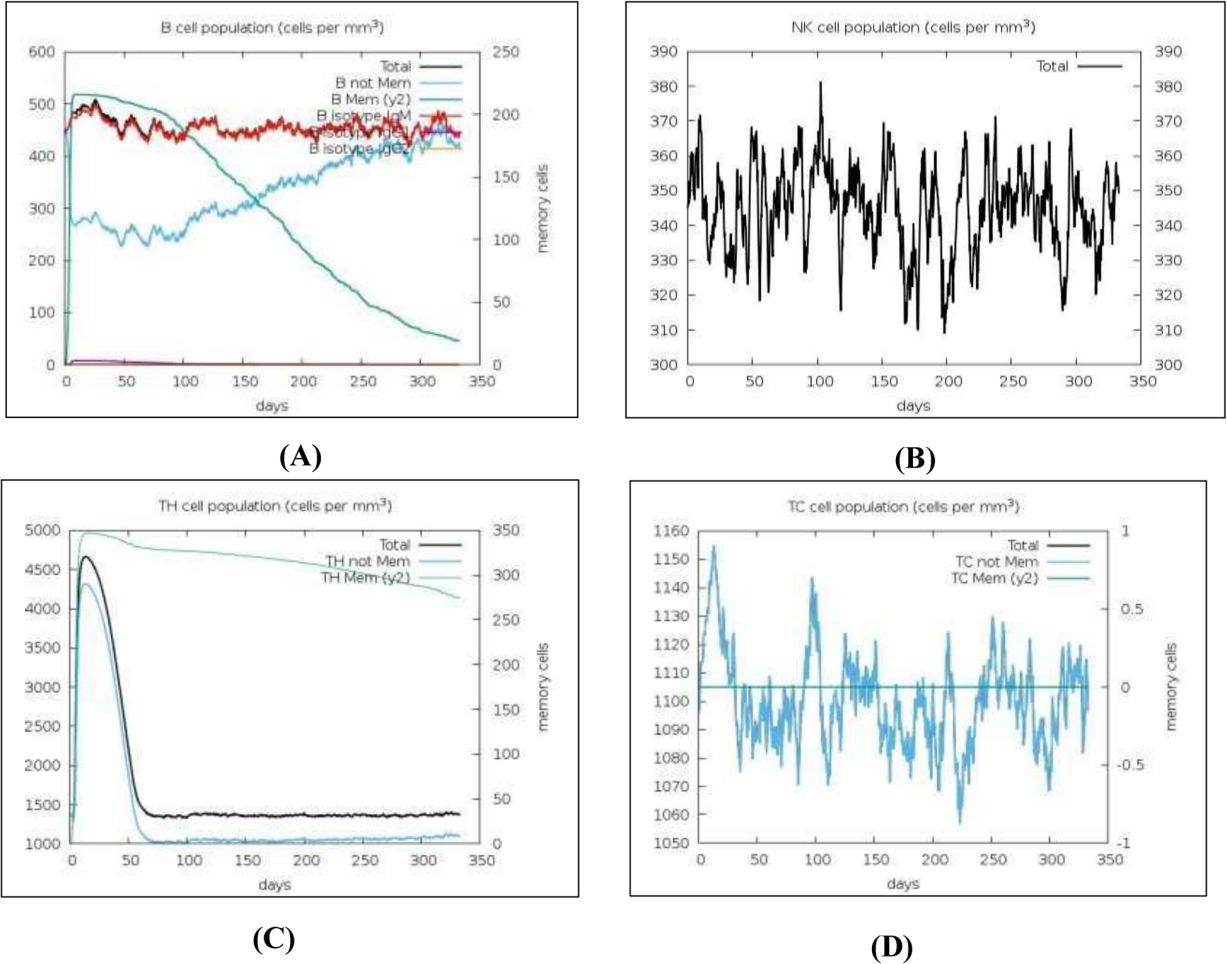
Production of immune cells against the antigenic protein. (**A**) B-cell population permm^3^. (**B**) NK cell population per mm^3^. (**C**) T_H_-cell population per mm^3^. (**D**) T_C_-cell population per mm^3^

Figure 6 illustrates a graph demonstrating the proliferation of immune cells and their concentration in mm^3^. The graph demonstrates the consistency of IgM-releasing B-cells and memory B-cells which persisted at high peaks for almost 60 days and steadily declined to 100 cells/mm^3^ on day 300. Furthermore, helper T-cells peaked within 35 days and remained almost consistent for 350 days. This validates the vaccine construct’s ability to confer an immediate immune response along with lasting long-term immunity. The levels of NK cells and cytotoxic T-cells also remained at considerably high levels, further eliciting the vaccine construct’s efficacy in mediating immunity against MARV infection.

## Discussion

A vaccine should induce an infection-induced natural immunity after a long-lasting adaptive immunity. Previously reported epitope-based vaccine constructs against MARV are mostly based on surface-exposed viral proteins. As delineated in studies conducted on the efficacy of vaccines against the SARS-Cov-2 virus [24], a conspicuous correlation becomes apparent between elevated mutation rates and heightened pathogenicity of viral infection, accompanied by the concomitant attenuation of the long-term relevancy of vaccine constructs. Similarly, the high mutation rates reported in VP40 and NP-VP intergenic regions [2] might lead to a potential loss of antigenicity of vaccine constructs based on these proteins over a period of time. Hunegnaw R et al. [66] reported about clinical trial of a single-shot ChAd3-MARV vaccine on non-human primates resulting in production of IgG specific for MARV-GP, which is susceptible to moderate levels of mutations [2] and thereby, potentially impinges on the sustained efficacy of the vaccine in the long term. In our study, the predicted MHC-I- and MHC-II-restricted T-cell epitopes as well as B-cell epitopes for antigenic targets were screened based on two main parameters: antigenicity score and conservancy levels. The selected T-cell and B-cell epitopes had average antigenicity scores of > 0.5 and were almost 100% conserved in five reported strains of MARV, which signifies that the epitopes are highly antigenic and are highly conserved. Hence, the vaccine construct is capable to maintain its antigenic integrity, notwithstanding the elevated mutation rate of VP40 and VP35, as well as the moderate mutation rate of GP [23]. All these factors consolidate our work pioneering, as we employed epitopes encompassing conserved regions of five endogenous and exogenous viral proteins, namely, GP, VP24, VP35, VP40 and RdRp, which indicate towards long term stability and efficacy of the vaccine construct in mediating an immune response against MARV infection.

Epitopes used for the combinatorial vaccine construct were selected after further screening based on docking studies, hydrophobicity, allergenicity, and other parameters. The final modeled multi-epitope vaccine construct was found to be highly antigenic and non-allergenic. The modeled protein construct has 324 amino acids and an average molecular weight of 34 kDa and is globular in nature. A Ramachandran favored score of 97.8 %, C-score of 14, and Z-score of 7.98 further consolidate the structural confidence and stability of the protein. According to WHO guidelines, a suitable vaccine candidate should have less than 1 transmembrane helices. Our designed vaccine construct was devoid of any transmembrane helix, as predicted using the DeepTMHMM server. This suggests ease of expression and purification of the protein vaccine. The estimated half-life in mammalian reticulocytes was 30 h, which is sufficient for generating an immune response. Hence, the results demonstrate that it is a strong antigenic vaccine protein that can efficiently stimulate innate immunity.

To analyze the molecular interaction of vaccine construct MarVax, molecular docking was carried out with TLR 2, 4, and 5. TLRs 2 and 5 are surface-exposed and recognize various PAMPs, and TLR4 plays an important role in amplification of inflammatory response and lipopolysaccharide recognition. Hence, interactions with these TLRs can indicate a potential offset of a stable inflammatory response. The interaction of the vaccine construct with TLR 5 had the strongest binding score of − 20.8 kcal/mol due to hydrogen bond formations between eight residues. The binding energy was moderately compared to the previously modeled vaccines [67], which might elicit a higher affinity and efficiency in mediating immune response.

The final molecular dynamics simulation showed that the protein, upon interaction with the cell surface TLRs, maintains its stability over time. Immune simulation-predicted immune responses of the host body towards the antigenic protein revealed the production of antibodies, memory B-cells, cytokines, and other immune cells within 2 weeks of vaccination, with antibody-releasing B-cells persisting for almost 60 days following the first vaccine. This further indicated that the vaccine molecule MarVax confers stable long-term immunity against the viral infection and is effective against different strains of virus due to the high conservancy of epitopes. This protein’s immunological simulation study provided further evidence that it has the potential to be a stable and robust antigenic vaccine protein to combat the deadly infection. Hence, based on various physicochemical and molecular dynamic interaction-based studies, we can say that the designed multi-epitope protein construct MarVax can be a stable, specific, and antigenic vaccine against the Marburg virus, which needs to be further consolidated by in vitro and in vivo studies for approval.

## Conclusion

Recurring outbreaks and fatality rates going as high as 88% have put Marburg virus disease at the forefront of research for the cure against the viral infection. Categorization of MARV as a priority pathogen A by The National Institute of Allergy and Infectious Diseases (NIAID) and category A bioterrorism agent by the Centers for Disease Control and Prevention (CDC) further necessitate the commercialization of vaccine and therapy against MARV infection. While previous studies have predicted multi-epitope vaccines, a vast majority of them do not address the long-term antigenicity of the vaccine construct, which might be affected owing to high mutation rates in certain viral proteins.

The multi-epitope vaccine designed by us combines epitopes from GP, VP24, VP35, VP40, and RdRp and is highly antigenic and immunogenic. Docking studies and results exhibited by molecular dynamics establish the vaccine construct as a stable molecule with higher affinity as compared to predecessors. The immune simulation of this vaccine revealed a strong immune response with significant immune cell and cytokine secretion within 2 weeks after vaccination, and the immune system being active for up to 60 days afterward. The population of memory B-cells, helper T-cells, and NK cells also remained stable for up to 350 days, establishing the vaccine construct’s efficacy in generating long-term stable immunity. Hence, the designed multi-epitope MarVax shows a strong potential to be an effective vaccine against MARV and is suitable for in vitro and in vivo validation.

### Supplementary Information

**Additional file 1: Supplementary Figure 1.** Epitope distribution in the modeled multi-epitope vaccine. B-cell and T-cell epitopes are highlighted in red and blue color, respectively. **Supplementary Figure 2.** Protein-TLR2 interaction. TLR is highlighted in green color, side chain in yellow while protein in cyan color. **Supplementary Figure 3.** Protein-TLR4 interaction. TLR is highlighted in green color, side chain in yellow while protein in cyan color. **Supplementary Figure 4.** Ramachandran plot of vaccine construct molecule, validates using the PROCHECK server **Supplementary Figure 5.** Z-score plot of vaccine construct molecule.

**Additional file 2: Supplementary Table 1.** T-cell MHC class I epitopes. **Supplementary Table 2.** T-cell MHC class II epitopes. **Supplementary Table 3.** B-cell epitopes.

## Abbreviations

MVD: Marburg virus disease
MARV: Marburg virus
EBOV: Ebola virus
GP: Glycoprotein
NP: Nucleoprotein
VP: Viral protein
RdRp: RNA-dependent RNA polymerase
TLR: Toll-like receptor
CTL: Cytotoxic T lymphocyte
HTL: Helper T lymphocyte
IEDB: Immune epitope database
PDB: Protein Data Bank
MD: Molecular dynamics
RMSD: Root mean square deviation
SASA: Solvent-accessible surface area
RMSF: Root mean square fluctuation
