# Supplementary material for: Designing a novel and combinatorial multi-antigenic epitope-based vaccine “MarVax” against Marburg virus—a reverse vaccinology and immunoinformatics approach
Source: J Genet Eng Biotechnol. 2023 Nov 28;21:143. doi: 10.1186/s43141-023-00575-w (PMC10681968; doi:10.1186/s43141-023-00575-w)

Supplementary Figures

Supplementary Figure 1: Epitope distribution in the modeled multi-epitope vaccine. B-cell and T-cell epitopes are highlighted in red and blue color respectively.

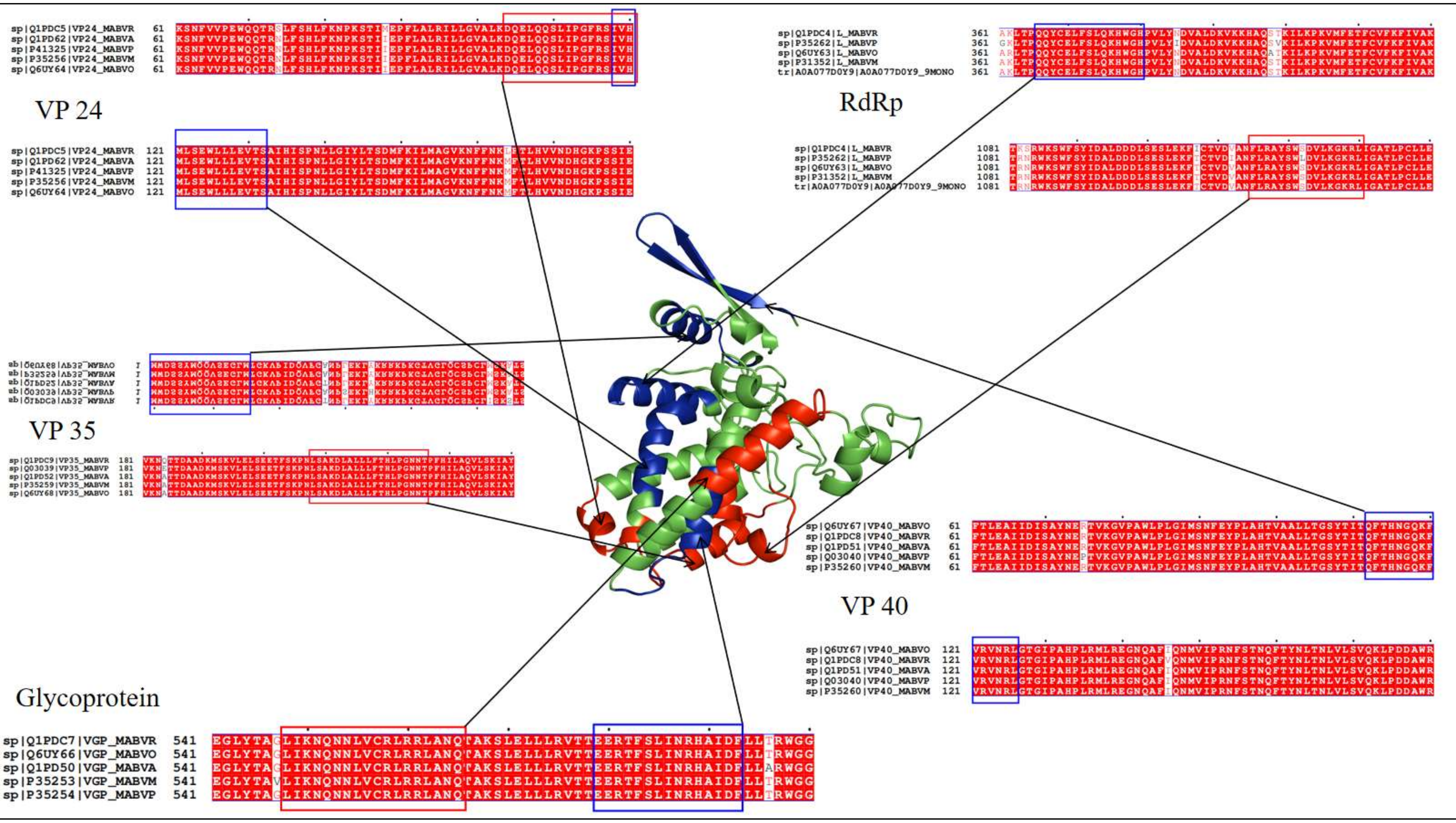

Supplementary Figure 2a: Protein-TLR2 interaction. TLR is highlighted in green colour, side chain in yellow while Protein in cyan colour.

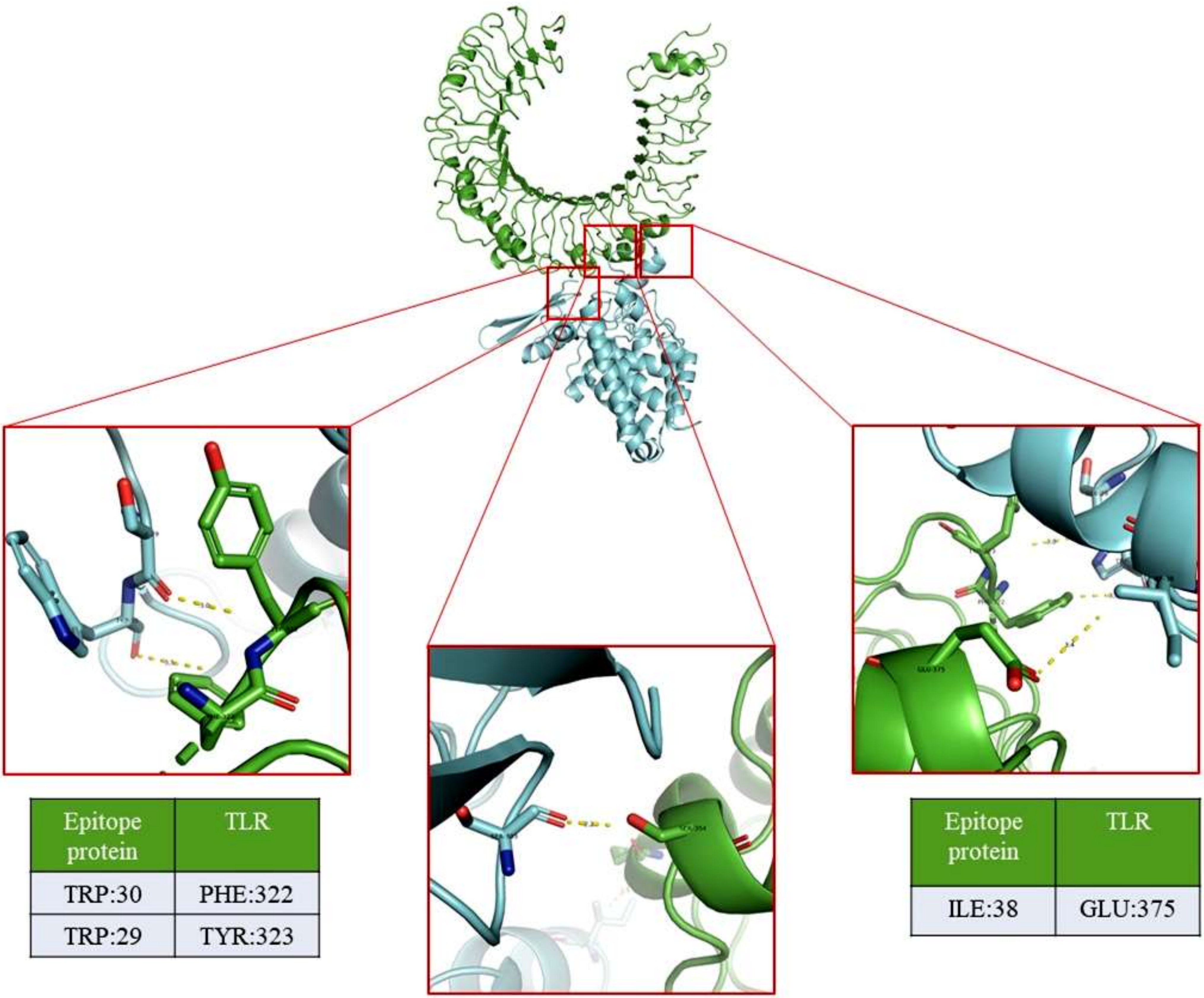

Epitope protein	TLR
TRP:30	PHE:322
TRP:29	TYR:323

Epitope protein	TLR
SER:303	SER:354

Epitope protein	TLR
ILE:38	GLU:375

Supplementary Figure 2b: Protein-TLR4 interaction. TLR is highlighted in green colour, side chain in yellow while Protein in cyan colour.

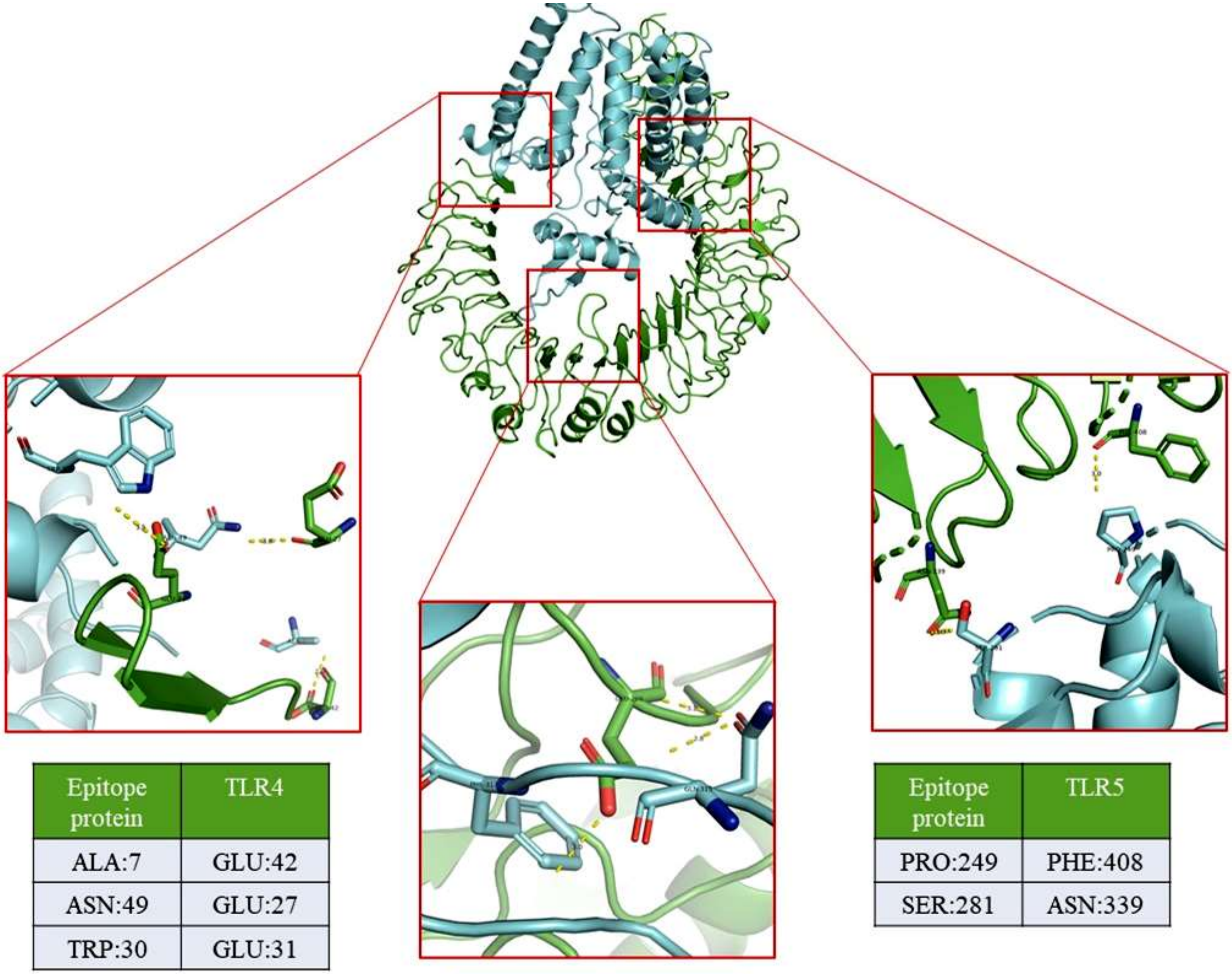

Epitope protein	TLR4
ALA:7	GLU:42
ASN:49	GLU:27
TRP:30	GLU:31

Epitope protein	TLR4
GLN:315	GLU:266
PHE:317	GLU:266

Epitope protein	TLR5
PRO:249	PHE:408
SER:281	ASN:339

Supplementary Figure 3a: Ramachandran Plot of vaccine construct molecule, validates using PROCHECK server

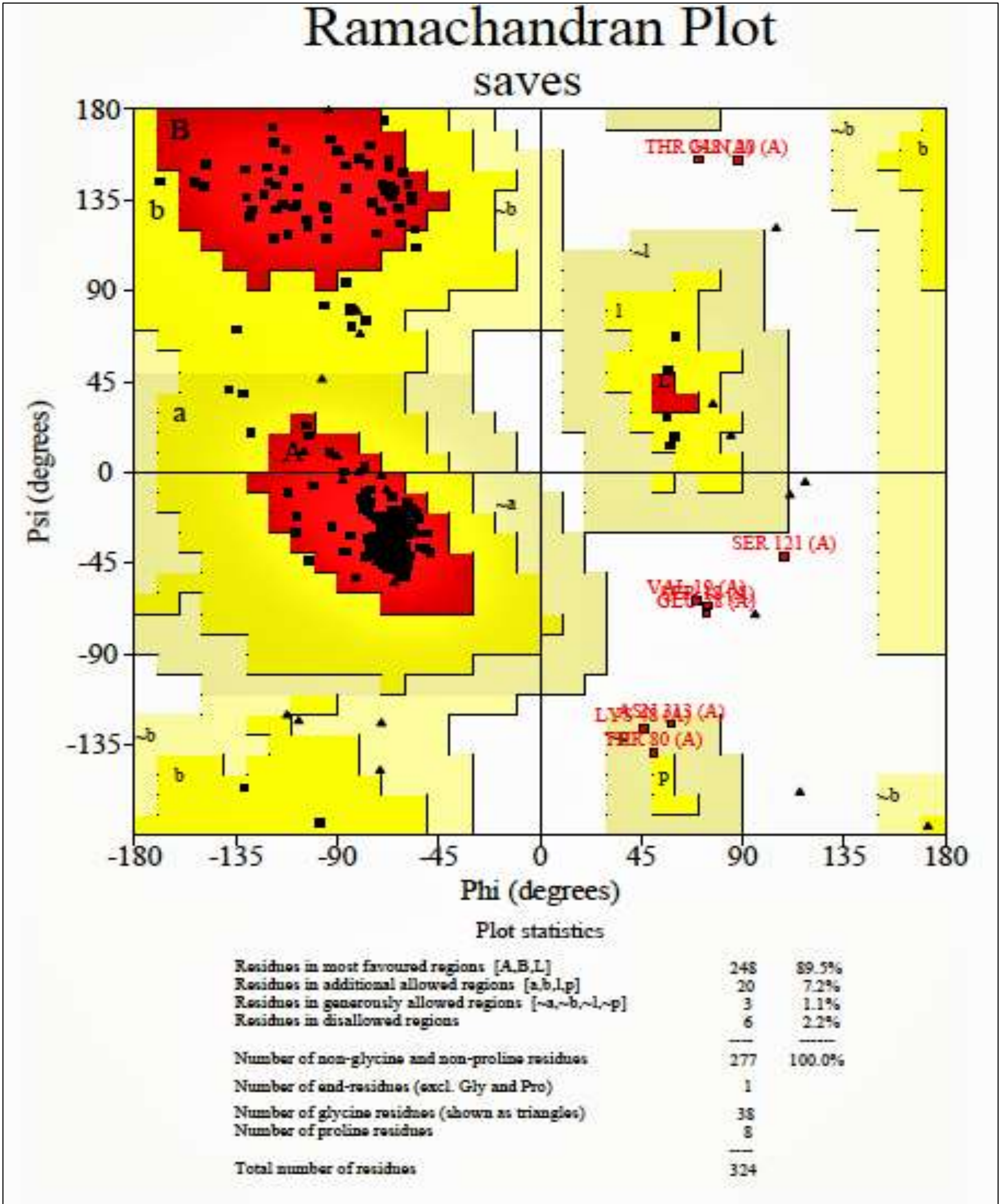

Supplementary Figure 3b: Z-score Plot of vaccine construct molecule

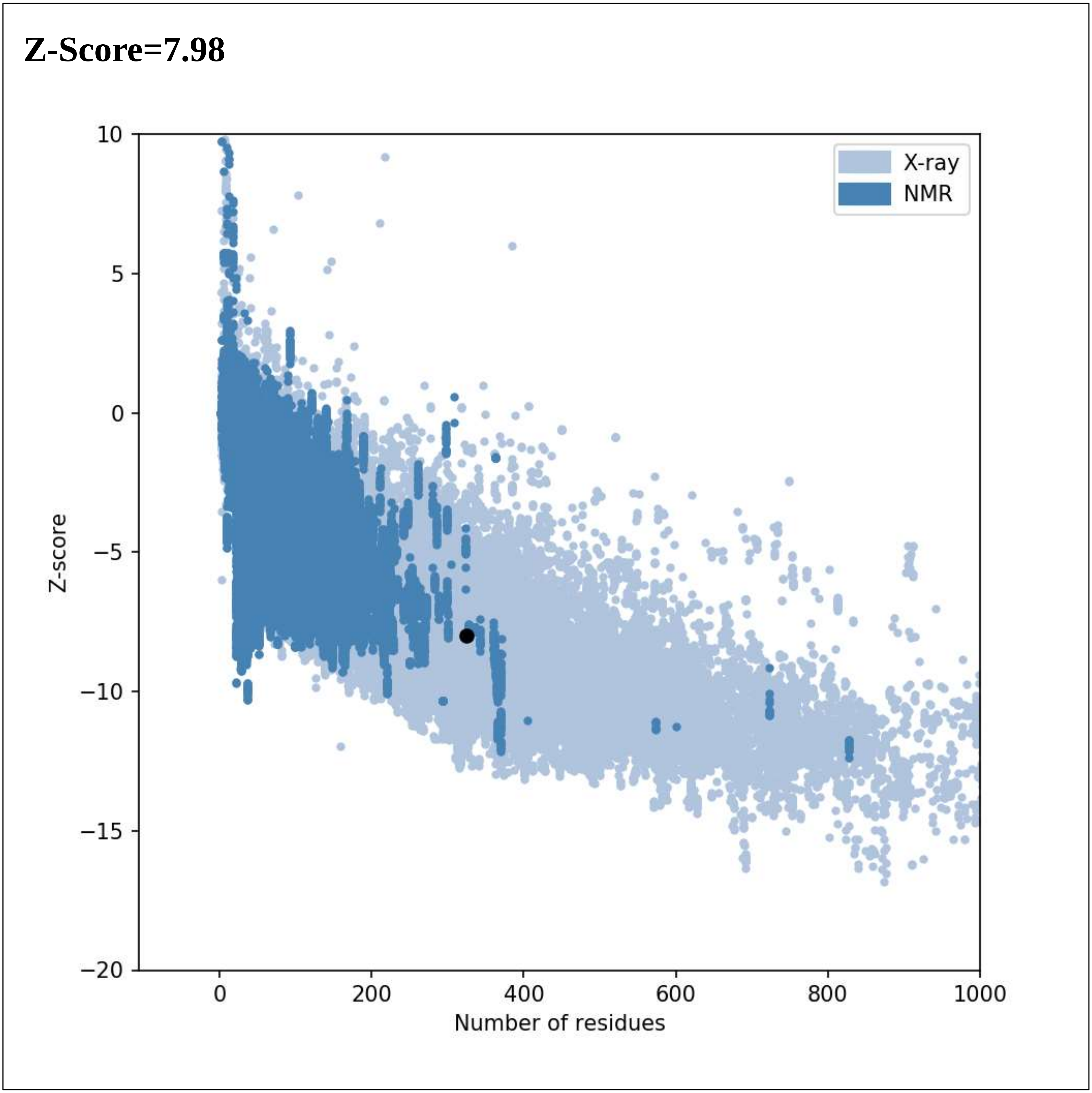

Supplement: Supplementary file 1 — Additional file 1: Supplementary Figure 1. Epitope distribution in the modeled multi-epitope vaccine. B-cell and T-cell epitopes are highlighted in red and blue color, respectively. Supplementary Figure 2. Protein-TLR2 interaction. TLR is highlighted in green color, side chain in yellow while protein in cyan color. Supplementary Figure 3. Protein-TLR4 interaction. TLR is highlighted in green color, side chain in yellow while protein in cyan color. Supplementary Figure 4. Ramachandran plot of vaccine construct molecule, validates using the PROCHECK server Supplementary Figure 5. Z-score plot of vaccine construct molecule. [file 43141_2023_575_MOESM1_ESM.pdf]
