# Supplementary material for: Designing a novel and combinatorial multi-antigenic epitope-based vaccine “MarVax” against Marburg virus—a reverse vaccinology and immunoinformatics approach
Source: J Genet Eng Biotechnol. 2023 Nov 28;21:143. doi: 10.1186/s43141-023-00575-w (PMC10681968; doi:10.1186/s43141-023-00575-w)
Supplement: Supplementary file 2 — Additional file 2: Supplementary Table 1. T-cell MHC class I epitopes. Supplementary Table 2. T-cell MHC class II epitopes. Supplementary Table 3. B-cell epitopes. [file 43141_2023_575_MOESM2_ESM.docx]

**Supplementary Table 1: T-Cell MHC Class I Epitopes**

| **Glycoprotein** | **RDRP** | **VP24** | **VP35** | **VP40** |
| --- | --- | --- | --- | --- |
| ^561^RLANQTAKSL^570^ | ^1565^EKSSSDAVRLF^1575^ | ^134^IHISPNLLGI^143^ | ^209^SAKDLALLLF^218^ | ^4^SSNYNTYMQYL^14^ |
| ^84^TEGEEAKTCY^89^ | ^814^QLPQSLKTM^822^ | ^115^FRSIVHMLSEW^125^ | ^155^HGVPPPQPAIF^165^ | ^198^HPNLPPIVLPT^208^ |
| ^511^DCDAELRIW^516^ | ^917^FLNPEKLFYR^926^ | ^101^ALKDQELQQSL^111^ | ^141^TTAPAAAFDAY^151^ | ^150^VIPRNFSTNQF^160^ |
| ^49^LMGFTLSGQK^58^ | ^814^QLPQSLKTM^822^ | ^31^KEPSVGGWTV^40^ | ^228^FHILAQVLSK^237^ | ^93^EYPLAHTVAAL^103^ |
| ^535^FFGPGIEGLY^544^ | ^777^VELAITTGY7^85^ | ^151^KILMAGVKNF^160^ | ^185^TTDAADKMSK^194^ | ^34^SNQQGITPNY^43^ |

**Supplementary Table 2: T-cell MHC Class II Epitopes**

| **Glycoprotein** | **RDRP** | **VP24** | **VP35** | **VP40** |
| --- | --- | --- | --- | --- |
| 579EERTFSLINRHAIDF^593^ | ^463^EWYFVEHEPLFSTKI^477^ | ^193^VNMGFLVEVRRIDIE^207^ | ^58^TDDIIWDQLIVKRTL^72^ | ^97^AHTVAALLTGSYTIT^111^ |
| ^641^GGKWWTSDWGVLTNL^655^ | ^725^AQITLVELKTKLKLK^739^ | ^108^QQSLIPGFRSIVHML^122^ | ^224^NNTPFHILAQVLSKI^238^ | ^55^GNVCHAFTLEAIIDI^69^ |
| ^521^VQEDDLAAGLSWIPF^535^ | ^366^QQYCELFSLQKHWGH^380^ | ^118^IVHMLSEWLLLEVTS^132^ | ^1^MWDSSYMQQVSEGLM^15^ | ^223^GPLLAISGILHQLRV^237^ |
| ^657^ILLLLSIAVLIALSC^671^ | ^147^WGMLLLVHLSQLARR^161^ | ^124^EWLLLEVTSAIHISP^138^ | ^192^MSKVLELSEETFSKP^206^ | ^112^QFTHNGQKFVRVNRL^126^ |
| ^77^PPKNVEYTEGEEAKT^91^ | ^730^VELKTKLKLKSSVMG^744^ | TSAIHISPNLLGIYL^145131^ | ^315^CVYSSEQGETRALKI^329^ | ^158^NQFTYNLTNLVLSVQ^172^ |

**Supplementary Table 3: B-cell Epitopes**

| **Glycoprotein** | **RdRp** | **VP24** | **VP40** | **VP35** |
| --- | --- | --- | --- | --- |
| ^546^AGLIKNQNNLVCRLRRL^562^ | ^232^VSVASYECFIMIKDVF^247^ | ^202^RRIDIEPETVLSESV^219^ | ^67^IDISAYNERTVKGV^80^ | ^318^SSEQGETRALKI^329^ |
| ^37^CSGTLQKTEDVH^48^ | ^1958^LLLKWKETDY^1967^ | ^104^DQELQQSLIPGFRSIVHM^121^ | ^151^IPRNFNQFTYN^163^ | ^208^LSAKDLALLLFTHLPGNNT^226^ |
| ^104^SLLLDPPSNIRDYPKCKT^121^ | ^1115^FLRAYSWSDVLKGKRL^1130^ | ^58^HHLKSNFVVP^67^ | ^67^IDISNERTVKGV^80^ | ^236^SKIAYKSGKSGAFLD^250^ |
| ^548^LIKNQNNLVCRLRRLANQ^565^ | ^1386^LYFDKPLDVDLNKYMD^1401^ | ^70^QQTRNLFSHLFKNPKST^86^ | ^248^RISLPADMFSVGMMSPV^272^ | ^301^RAVPPNPTIDKGW^313^ |
| ^39^GTLQKTEDV^47^ | ^2026^LDSVIQYLPEDSDILTMD^2043^ | ^70^AELSTRYNLPALDLNSTARW^86^ | ^110^ITQFTHNGQKFVR^122^ | ^316^VYSSEQGETR^325^ |
